# COVID-19 Crisis Management: Lessons From the United Arab Emirates Leaders

**DOI:** 10.3389/fpubh.2021.724494

**Published:** 2021-10-29

**Authors:** Walid Abbas Zaher, Faheem Ahamed, Subhashini Ganesan, Katherine Warren, Ashish Koshy

**Affiliations:** ^1^Research Team, G42 Healthcare, Abu Dhabi, United Arab Emirates; ^2^Marketing Team, G42, Abu Dhabi, United Arab Emirates; ^3^G42 Healthcare, Abu Dhabi, United Arab Emirates

**Keywords:** leadership, COVID-19, healthcare, crisis management, UAE

## Abstract

This study analyses the UAE leadership's approach in response to the COVID-19 crisis through the Organization for Economic Co-operation and Development's Strategic Crisis Management Framework. This framework analyzes the crisis management in three phases: the preparedness, the response to mitigate damage and the feedback mechanism after the crisis. The analysis showed that the key components of the UAE's crisis management included efficient and able governance, integrated utilization of public-private partnerships and a global workforce of excellence. As a result, the UAE now ranks among the top 10 countries worldwide for its leadership and proactive approach during the COVID-19 pandemic, according to Global Response to Infectious Diseases Index. The SWOT analysis on the response toward COVID-19 crisis management helped in critically analyzing and understanding the UAE's unified and systematic response to the pandemic, which provides developing and developed countries alike a new high standard for leadership and effective public health management.

## Introduction

Ever since it has been identified in Wuhan in the Hubei province in China in December of 2019, the new strain of coronavirus responsible for the coronavirus disease 2019 or COVID-19 has spread to 213 countries and territories around the world with over 240M+ reported confirmed cases and a death toll of over 3.3 million lives (1, 2). More than a year into the pandemic, many countries and their leaders are being hailed among the most efficient and effective in managing the COVID-19 crisis, while others have been called out for failure to act in a timely and responsible manner leading to tragically avoidable deaths and loss of faith in those countries' leadership and the government's ability to contain the outbreak successfully.

With globalization, international interconnectedness, and interdependence through global supply chains, as well as the movement of people, goods, knowledge, and infectious diseases, modern leaders must consider not only the primary impact on their countries' population, economy, and infrastructure, but also the secondary impact that may result from crisis experienced outside of their borders (3). It is of paramount importance that governments consider their idiosyncratic circumstances and work with them before, during, and after a crisis in a manner that utilizes expertise and competencies thereby maximizing benefit and reducing the burden on the populations (3, 4).

The UAE population is 9.99 million of which over 88% are expatriates belonging to more than 200 nationalities (5). Health service delivery in the UAE is through public-private partnerships and is regulated at both federal and local (emirate) levels. This is overseen by the Ministry of Health and Prevention (MOHAP) in the northern emirates, the Abu Dhabi Department of Health (DoH) and the Abu Dhabi Health Services Company (SEHA) in Abu Dhabi, and the Dubai Health Authority (DHA) in Dubai (6). In addition, the UAE has created two specialized healthcare free zones, the Dubai Healthcare City regulated by the Dubai Healthcare City Authority and Sharjah Healthcare City, aimed at attracting international medical service providers to set up in a 100% ownership structure and encourage medical tourism (7, 8).

Further, the UAE has an established National Emergency, Crisis, and Disaster Management Authority (NCEMA) the main aim of which is to regulate and coordinate all emergency and crisis management. In this paper, we will examine the UAE's response to the COVID-19 pandemic and crisis management measures taken by its leaders to limit and mitigate the effects of the COVID-19 disease.

The UAE leadership's response and handling of the COVID-19 crisis was examined through the lens of the Organization for Economic Co-operation and Development's (OECD) Strategic Crisis Management Framework (3). The outcomes of national efforts was analyzed using a set of metrics for efficacy and effect. Finally, a SWOT analysis on the response measures was done to critically analyze and understand the key areas affecting the pandemic management in the UAE.

## UAE Leadership and Crisis Management in the COVID-19 Pandemic

### The OECD Strategic Crisis Management Framework

After examining several crisis management frameworks, we opted to use that of the Organization for Economic Co-operation and Development (OECD) (3) because it was designed to serve as a guide on a country level rather than organizational and because it provides a valuable set of metrics to evaluate response systems, including policy influence. The OECD considers multiple aspects for the changing face of successful governance during a crisis, including the increased demands and expectations of modern-day citizens and the media. The three main phases of the OECD are:

1. Preparedness before a crisis and it involves activities aimed at developing capacities that will assist in effective anticipation, response, and recovery from a crisis.
2. Response to limit damages once the crisis occurs.
3. Clear feedback after the crisis through feedback mechanisms that review, analyze, and draw lessons from the actions taken to mitigate the damage.

Each of these components encompasses a set of requirements and practices that will be applied to the UAE to judge its level of success in tackling the COVID-19 pandemic nationally, regionally, and internationally.

#### Preparedness Before a Crisis

According to the OECD Strategic Crisis Management Framework, the elements of the crisis management preparedness phase are risk assessment of threats and vulnerabilities, developing early detection and warning systems, storage and maintenance of adequate equipment and supplies, regular training drills, adequate institutional structures supported by a robust legislative and policy development system, and the allocation of resources through budgets.

In terms of preparedness to manage an outbreak and according to the 2019 first Global Health Security Index (GHSI), the UAE placed 25^th^ among 195 countries in commitments to improving national capacity, financing, and adherence to norms and 56^th^ in overall preparedness with a score of 46.7 (9).

Economically and according to the World Economic Forum's 2019 edition of the Global Competitiveness Report, which covered 141 economies that account for 99% of the world's Gross Domestic Product (GDP), the UAE ranked 25^th^ in the world (gaining two positions since the last edition of the report) and second in the MENA region (10). The main competitive advantages of the UAE cited in this report are its stable macroeconomic environment, a sound product market and infrastructure, and one of the most modern transport systems in the world.

In terms of crisis and disaster management, the UAE established the National Emergency, Crisis, and Disaster Management Authority (NCEMA) in 2012. The NCEMA works under the umbrella and supervision of the Supreme Council for National Security. It is the main national body responsible for regulating and coordinating all efforts of emergency, crisis, and disaster management, as well as the development of a national plan for responding to emergencies (11). Also, the UAE hosted and participated in the United Nations Office for Disaster Risk Reduction's workshop on understanding the Sendai Framework for Disaster Risk Reduction in October 2017, which aims at enhancing a risk reduction environment by emphasizing the role of the State in achieving its disaster reduction goals (4).

While the UAE had developed disaster response systems, the pandemic of international crisis of this magnitude was unanticipated, and the country was as naïve as rest of the world was. But what differed was its dynamic and aggressive response allowing for the rapid development and expansion of systems once the disease challenge made itself present. This allowed the country to immediately begin and scale-up response protocols that allowed for wide-scale testing, surveillance, clinical trial development, international collaboration, and vaccine dissemination.

#### Response to Limit Damages

##### Crisis Monitoring

The first step in responding to an unexpected crisis according to the OECD crisis management framework is to identify swiftly and effectively the source of the crisis, often requiring integration of scientific expertise to break down a complex situation into simpler scientific or technical components (3). The first confirmed cases of COVID-19 in the UAE and the Gulf region were reported by the Ministry of Health and Prevention on January 29, 2020 (12, 13). Two days later on January 31^st^, 2020, the Government announced that all suspected and confirmed cases of COVID-19 are to be treated as emergencies and that medical care for all COVID-19 patients was to be provided free of charge (12). Decisive and prompt responses of this kind from the UAE government right from the early stages of the pandemic likely contributed to the diminished early burden of the disease on the country (14, 15). From school closures and suspension of flights to drive through testing centers and national sterilization and cleaning protocols, the first 100 days in the UAE's response to the COVID-19 pandemic were marked by multiple successful and considered COVID-19 response measures and mandates (16–19) (Table 1).

**Table 1:** The proactive measures taken by the UAE leadership and NCEMA to limit the spread of the coronavirus disease.

**Date**	**Measures taken**
8/3/2020	Closure of all schools and higher education institutions.
15/3/2020	Remote working was activated for two wk for select categories of public sector employees in the federal authorities.
17/3/2020	Retailers reduced opening hours from midday to 8pm only - as opposed to their regular 10am to 10pm timings
22/3/2020	Students began virtual classes.
24/3/2020	Sheik Hamdan bin Mohammed bin Rashid Al Maktoum, Crown Prince of Dubai and Chairman of the Dubai Executive Council launched the Day for Dubai app, as part of the 'Your City Needs You' campaign. The initiative called on Emiratis and expats alike to help the country's various teams working to protect the community.
25/3/2020	- All fresh food markets and commercial centers, including shopping malls, directed to close for two wk. Pharmacies, and food retail outlets (including cooperative societies, groceries, and supermarkets) were allowed to stay open. Restaurants were directed to limit their services to deliveries only. - The National Emergency and Crisis and Disasters Management and the Civil Aviation Authority announced the suspension of all inbound and outbound passenger flights and the transit of airline passengers in the UAE for two wk. - Private companies and commercial establishments implemented remote-work system for 80 per cent of their workforce, as per government directives. - Prohibition of public gatherings until further notice.
26/3/2020	- UAE began a nationwide disinfection program to sanitize the country. - An online permit system was launched in Dubai, through which residents could request permission to leave their homes for essential work or purposes. Abu Dhabi and Sharjah also followed by calling on residents to apply for permits if they wanted to step out of their homes.
27/3/2020	Dubai Police activated radars to monitor motorists violating quarantine measures during the National Sterilization Program.
28/3/2020	SEHA launches first drive-through screening for COVID-19 at Zayed Sports City.
1/4/2020	The Ministry of Human Resources and Emiratization issued a new remote work policy for private businesses. Under the policy, office-based workers must be limited to 30 per cent of the company's workforce.
4/4/2020	Supreme Committee of Crisis and Disaster Management, in coordination with the Command and Control Center for Combating COVID-19 announce a two-wk 24-h sterilization campaign. Residents were directed to wear face masks and gloves at all times outside the home, and follow social distancing measures. Authorities noted they would enforce strict restrictions on residents' movements during this time.
5/4/2020	- 'Early Leave' initiative launched to enable all residents in the private sector who wish to return to their home countries to do so. - Roads and Transport Authority of Dubai also suspended metro, tram, and intercity bus operations until further notice.
7/4/2020	- Dubai Economy announced that all commercial activities except for vital and support sectors, would remain closed until further notice. - The Dubai Health Authority announced it has opened its first drive-through Covid-19 testing center at the Al Nasr Club for the public.
9/4/2020	SEHA opens 13 additional drive-through COVID-19 testing centers.
6/2020	Gradual reopening of UAE Economy
16/6/2020	MOHAP hosts world's first Phase III clinical trials of an inactivated vaccine to combat COVID-19
16/9/2020	UAE authorizes emergency use of COVID-19 vaccine for frontline health workers
14/12/2020	Vaccination launched for the residents to get free vaccination
9/1/2021	More than 1 million people vaccinated
17/3/2021	More than 50 % of the UAE population vaccinated
29/3/2021	Abu Dhabi launched new COVID-19 vaccine plant with China's Sinopharm to manufacture inactivated Sinopharm vaccine in the UAE (Hayat-Vax)
29/5/2021	Free Sinopharm vaccine booster dose to those eligible
2/8/2021	UAE approves Sinopharm COVID-19 vaccine for children 3-17 years
3/8/2021	UAE among first countries of the world to receive Sotrovimab medicine for treatment of COVID-19
29/8/2021	Reopening of schools with full capacity.
30/8/2021	Reduction in prices of COVID-19 tests
31/8/2021	- COVID-19 infections down by 62% compared to January 2021 - More than 76% of the population fully vaccinated

##### Managing the Emergency Response Network

The OECD Strategic Crisis Management Framework also draws attention to the importance of effective management of the emergency response network due to the need of critical decision making as the crisis develops and the involvement of the civil society, including volunteer organizations and non-governmental organizations (NGOs) (3). The two main official bodies tasked with the response to the pandemic and coordination of the national efforts across the various sectors are the National Emergency, Crisis, and Disaster Management Authority (NCEMA) and the COVID-19 Command and Control Center (CCC). The CCC interfaces with the police, state security, ambulance services, the municipality, private health operators, healthcare academics, epidemiologists, and volunteers to classify patients and prioritize the provision of treatment where most required (20).

Realizing the role technology can play in limiting the spread of the disease, the UAE moved to quickly adopt telehealth and teleconsultation services (21). The existing advanced technology and communications infrastructure and the regulatory authorities' agility facilitated the development and enforcement of relevant policies as required by the rapidly changing circumstances and needs. Telehealth serves as an important tool in flattening the infection curve and mitigating the impact of the COVID-19 crisis because it limits human-to-human contact, particularly reducing nosocomial infections (22). To increase tracking and tracing efforts, NCEMA launched Al Hosn UAE App for collective contact tracing of COVID-19 patients and those who come in contact with them in early 2020 (23, 24).

COVID-19 volunteer programs across the UAE were also launched by local and federal authorities and had a marked effect in easing the burden and impact of the pandemic. These volunteer programs were all supervised and coordinated by the Supreme National Committee for Volunteerism during Crises, which was also responsible for governing volunteers' health and safety (24).

In addition to national effort, the United Arab Emirates made substantial humanitarian efforts to extend support to countries fighting the coronavirus disease both regionally and globally by working with international bodies such as the United Nations and the World Health Organization. This included extending medical aid, supplies, and testing capacities to Iran, African countries, and Cuba (25–29).

##### Leadership Through Communication

The OECD framework stresses the importance of leadership with high adaptive capacities during crisis management especially for communication, maintaining trust in the government, and “meaning-making”. During turbulent times, leaders who are able to maintain the trust of the governed populations and maintain a reputation of competence play an important role in reducing anxiety in the populace and maintaining effective responses (30). Effective communication through the appropriate channels during a crisis is paramount to raise awareness among members of the population, communicate risks, engage the community, and to counter the spread of misinformation and bad practices (3). The UAE ensured transparency with its population on the COVID-19 situation by disseminating daily statistics, including total number of cases, number of deaths, number of tests performed, total number of recoveries, and up-to-date health regulations. These statistics are made available through the Ministry of Health and Prevention's website, as well as the websites of the local health authorities, such as the Department of Health and the Abu Dhabi Health Services Company (SEHA) in Abu Dhabi and the Dubai Health Authority and Dubai Healthcare City Authority in Dubai. In addition, these authorities utilized social media platforms, including Twitter and Instagram, as well as official news outlets and traditional media to expand reach of the messaging and more widely disseminate information.

##### Vaccine & Technology Initiatives

An important part of this crisis management is bringing an end to the current pandemic. In the current scenario, effective vaccination is the only solution leading to eradication or long-term reduced transmission of this disease. This inevitable attention to the vaccines has made them the unexpected superheroes of 2020 (31).

The UAE leadership was proactive in running the first WHO-enlisted phase III clinical trial of an inactivated vaccine against SARS-CoV-2 in the UAE, through a partnership between Sinopharm's CNBG and Abu Dhabi-based Group 42 (G42) under the supervision of the Department of Health of Abu Dhabi (DOH) and UAE Ministry of Health and Prevention (MOHAP) (32). Thousands of volunteers from different nationalities registered and participated in the trial. It is a vivid example of national initiatives to foster population health and to enhance the UAE's medical research and development capabilities.

After the initial successful results of the trial in UAE, the use of the inactivated COVID-19 vaccine was approved for frontline workers, which was subsequently successfully administered to the cohort (33, 34). The vaccine is given free of cost for all residents of the UAE (35) and the country at the forefront, leading the global vaccination rate (36).

#### Feedback After the Crisis

According to the OECD framework, as the crisis ends, the government must signal to its people and encourage a return to normal. Then, after the crisis is officially over, an in-depth review and a multi-level analysis are to be conducted to assess the country's response to the crisis within, between, and across all the relevant agencies. Considering that the COVID-19 pandemic is still ongoing as of the date of writing this paper, a more encompassing review of the UAE's response and information integration will need to be completed in the future. However, there currently is evidence of consistent new information integration into the public health and disease control decision-making processes in the UAE (37). Moreover, the UAE has already developed a post-COVID-19 strategy for managing the impact of the pandemic spanning six sectors: health, education, economy, food security, society, and government (38).

As a pandemic of this magnitude was unprecedented for the whole world, the UAE had not been previously confronted with such a ranging public health crisis. However, the country was primed to be responsive to the dynamic needs of a rapidly evolving world through existing pipelines of innovation and economic growth. This was able to be leveraged to quickly respond to the viral disease challenge by rapidly engaging international collaborations and partnerships for technology, therapeutics, and vaccine implementation. The country also fostered and pushed the rapid development of multiple targeted response systems by growing widescale testing and sequencing facilities inside the country, allowing for a faster turnaround of results and thus augmented public health intervention. The new analytic capacity of the country allows for a high proportion of the population being tested and protected. The infrastructure built and expanded over 2020 into 2021 now allows for effective identification, sequencing, treatment, vaccination, and response for the current pandemic. Importantly, this robust infrastructure is now also in place for future outbreaks and public health challenges.

### Tangible Outcomes of the UAE Response to the COVID-19 Pandemic

#### Global Response to Infectious Diseases (GRID) Index

The United Arab Emirates (UAE) has been among the top 10 countries praised internationally for its leadership and proactive approach during the coronavirus disease outbreak according to GRID index (39). The criteria used to evaluate countries on how well the country is dealing with coronavirus include the number of tests per million of population, the number of deaths per case and per million of population, the number of cases per million of population, and the CP Index (the perceived transparency of a country). The percentage of cases tested compared to the population indicates how preemptive the healthcare system is at handling the pandemic. The number of deaths as a ratio of cases shows the efficiency of the overall healthcare system.

#### COVID-19 Screening Tests and Vaccination Coverage

In March 2020, the UAE succeeded in reaching one of the highest per-capita testing rates worldwide (40). It has conducted more than 200,000 tests per 100K population and has become the number one country in COVID-19 screening per capita, also it leads other developed countries in scale of testing compare to the scale of outbreak (41).

The country has one of the highest COVID-19 vaccination rates with more than 75% of the population fully vaccinated (42).

#### Deep Knowledge Group's (DKG) COVID-19 Regional Safety Assessment

The DKG used a subset of 20 parameters from a full pool of 130 qualitative and quantitative parameters (grouped into 6 broad and top-level categories) to “analyze and rank the economic, social and health stability achieved by 200 regions, countries and territories, as well as the strengths, weaknesses, opportunities, and threats or risks that they possess and face against the global health and economic crisis triggered by COVID-19” (43). According to the DKG report, the UAE scored 700 cumulative points and ranks among the Tier 1 countries (44). The report attributes this to the substantial investments made in the area of medical modernization and the development of cutting-edge healthcare technologies which is supported by figures on healthcare spending in the UAE which has grown at a Compound Annual Growth Rate of 8.8 percent *(0.8%) between 2011 and 2019 and is expected to reach $2.4 billion by 2025 and $3.6 billion by 2030 (45). In 2017, the UAE health expenditure per capita was 1,357 US dollars (45, 46), which is higher than the global average of 1,061 US dollars for the same year (47). The UAE has advanced to 8^th^ position globally in Bloomberg index of the most resilient countries that managed the coronavirus (COVID-19) pandemic (48).

### SWOT Analysis on Management of Pandemic in the UAE

**Figure 1:**
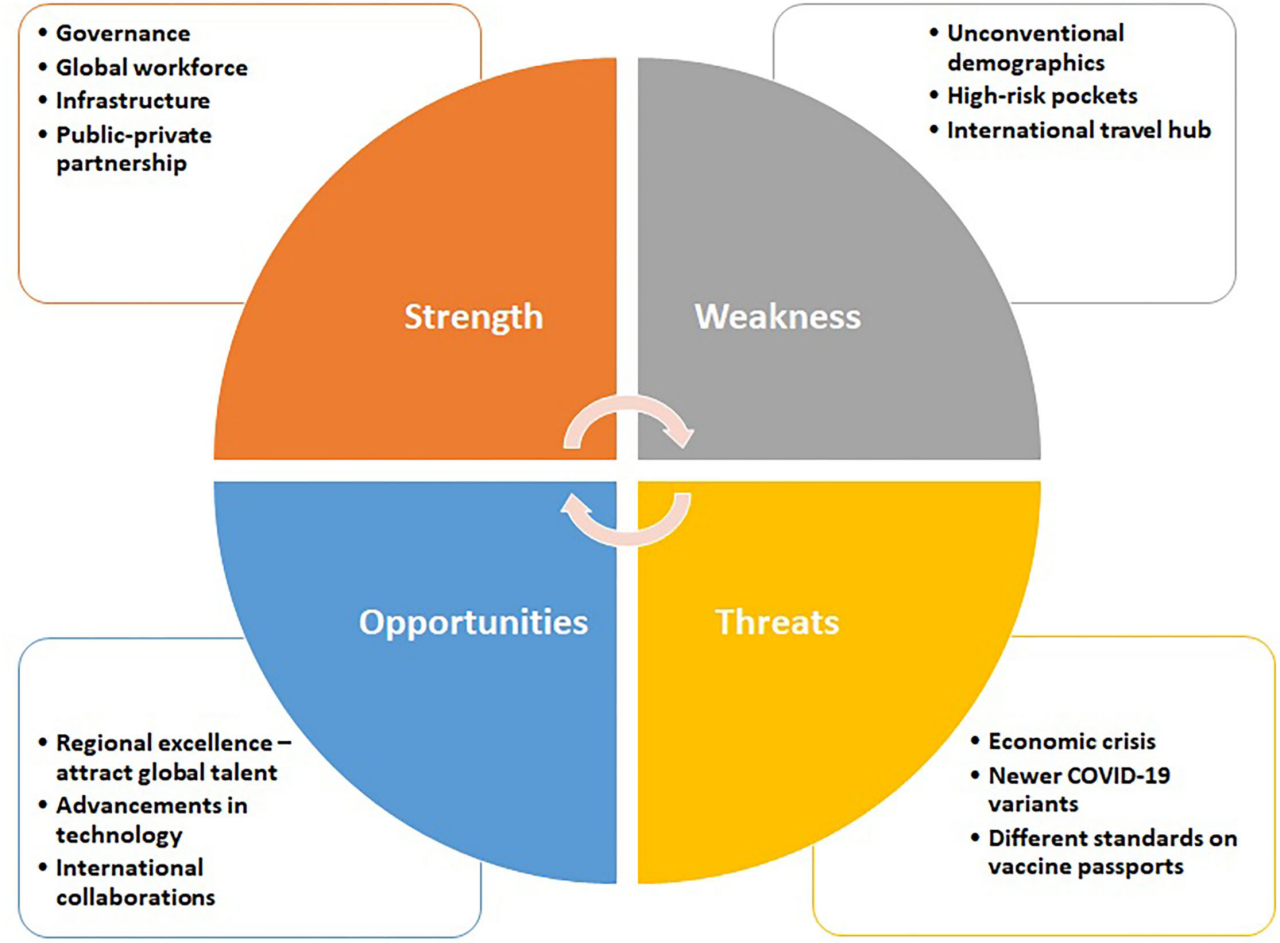
SWOT analysis on management of pandemic in the UAE.

A SWOT analysis on the COVID-19 crisis management in the UAE (Figure 1).

#### Strengths

The country has an existing nation-wide healthcare integrated system that is able to log and track infection on a personal and population-based scale, allowing for more nuanced and precise public health and preventative intervention. This is enhanced by the digitized management systems available that allow real time comprehension of health and disease status within the country and between the Emirates. The public-private partnerships model built in the healthcare services serves the backbone of this robust system between the government and health services with the private companies ranging from diagnostics to interventional services. This, alongside consolidated and effective governance, facilitates rapid and coordinated action across the populace. This coordination of effort extends to a global workforce that can interact across the inhabiting communities and visitors to the region. A final strength of the country lies within the mass, successful vaccine effort that rapidly covered a large portion of the populace and continues to cover the individuals in the country with ease of access to vaccination, high adherence rates, and effective, multiple-dose mandate campaigns.

#### Weakness

As much as the multicultural population dynamics is a strength to this nation, one of the major weaknesses in handling the COVID-19 situation comes from the difficulties inherent in such highly multicultural, expatriate communities that comprise the largest part of the population. This plays a key role in reducing effectiveness in response to disease control that differ between groups, with some more prone, locally, to lower adherence to pandemic guidelines and increased cohabitation density areas that create high-risk pockets of infection; this is particularly observed in in lower socioeconomic demographic groups due to living situations and population habits. The UAE also plays host to a large hub of international travel, particularly from regions within the Middle East, Indian subcontinent, Central Asia, and Africa, that have, at times, more challenged pandemic management infrastructures, preventative measure adherence, and vaccine availability. This increases the introductions of individuals from these populations into local communities by way of travel of the expatriates to home countries and international tourism to and through the UAE.

#### Opportunities

The UAE with the immense capacity for attracting global talent for the workforce, research, and development leverages this opportunity to build a robust ecosystem of knowledge and capability, as well as foster the creation of centers of excellence in the region. The UAE also attracts partnerships, collaborations, and development with leading companies across the world and has global connection networks that aid in the development and augmentation of technology and infrastructure within and beyond the health sector. Further these opportunities of development extend beyond the pandemic control and are utilized in the national drive toward personalized, proactive, and precision medical and healthcare systems and away from retroactive sick care.

#### Threats

The threats that face the UAE during this pandemic are particularly pronounced in magnitude due to the amorphous nature of the pandemic and the pressure on economic revival. The population of the United Arab Emirates comprises a multicultural collection of subpopulations with almost 88% expatriate population. This means that disease control, mitigation strategies, lifestyles, and responses need to be tailored to encompass a very diverse population set. The diversity of nationalities also increases the threat pressure because travel to and return from native countries serves as high potential for exposure to novel variants and disease transmission events. The UAE is also challenged by the emphasis on tourism, destination travel, and variable travel restrictions between Emirates and from across the globe that mandates a constant flux of travelers to maintain economic equilibrium. As above, this will increase exposure and introduction of a variety of diseases and variants. Further in the global politics, country wise different standards on vaccine passports, travel red lists, and bans make pandemic control even more hard-pressed.

## Conclusion

The COVID-19 pandemic has proven to be a true crisis that tested the strength of people, populations, and the leaders responsible for their care. The leadership of the UAE coordinated an integrated effort across the diverse sectors of the country, ranging from government to healthcare, private to public sector, and every day-to-emergency services and have set new high standard for leadership and effective public health management for countries around the world.

## Data Availability Statement

The original contributions presented in the study are included in the article/supplementary material, further inquiries can be directed to the corresponding author/s.

## Author Contributions

All authors have made substantial contribution to the conception of the work, collection of data, analysis, drafting the work, and revising it critically for important intellectual content.

## Conflict of Interest

Authors in the study were employed by G42 Healthcare. All authors declare that the research was conducted in the absence of any commercial or financial relationships that could be construed as a potential conflict of interest.

## Publisher's Note

All claims expressed in this article are solely those of the authors and do not necessarily represent those of their affiliated organizations, or those of the publisher, the editors and the reviewers. Any product that may be evaluated in this article, or claim that may be made by its manufacturer, is not guaranteed or endorsed by the publisher.
